# The Outer Pore and Selectivity Filter of TRPA1

**DOI:** 10.1371/journal.pone.0166167

**Published:** 2016-11-08

**Authors:** Adam P. Christensen, Nurunisa Akyuz, David P. Corey

**Affiliations:** 1 Program in Neuroscience, Harvard Medical School, 220 Longwood Ave, Boston, Massachusetts, United States of America; 2 Department of Neurobiology and Howard Hughes Medical Institute, Harvard Medical School, 220 Longwood Ave, Boston, Massachusetts, United States of America; Dalhousie University, CANADA

## Abstract

TRPA1 (transient-receptor-potential-related ion channel with ankyrin domains) is a direct receptor or indirect effector for a wide variety of nociceptive signals, and thus is a compelling target for development of analgesic pharmaceuticals such as channel blockers. Recently, the structure of TRPA1 was reported, providing insights into channel assembly and pore architecture. Here we report whole-cell and single-channel current recordings of wild-type human TRPA1 as well as TRPA1 bearing point mutations of key charged residues in the outer pore. These measurements demonstrate that the glutamate at position 920 plays an important role in collecting cations into the mouth of the pore, by changing the effective surface potential by ~16 mV, while acidic residues further out have little effect on permeation. Electrophysiology experiments also confirm that the aspartate residue at position 915 represents a constriction site of the TRPA1 pore and is critical in controlling ion permeation.

## Introduction

TRPA1, the only mammalian member of the TRPA subfamily of the transient receptor potential (TRP) ion channel family, is highly expressed in dorsal root and trigeminal ganglion neurons. There, it has a variety of sensory roles, especially sensation of painful or irritating stimuli [1–10]. A plethora of irritating chemicals activates TRPA1; these include mustard oil, cinnamaldehyde, allicin, eugenol, gingerol, and thymol (all from plant extracts) as well as the environmental irritants acrolein and formaldehyde [2, 4, 10–15]. TRPA1 is also activated by menthol (a cooling agent), 4-hydroxynonenal (a product of oxidative stress), tetrahydrocannabinol (a psychoactive component of marijuana), *N*-methylmaleimide (a cysteine-modifying reagent), Ca^2+^ ions, and alkalization [2, 12, 16–21]. Finally, it is downstream of receptors that activate the phospholipase C (PLC) pathway, such as the bradykinin receptor.

TRPA1 has six transmembrane domains with a reentrant loop between the fifth and sixth that creates the pathway for ion permeation (reviewed in [22]), and intracellular N- and C-termini [23]. Many irritating chemicals activate TRPA1 through covalent modification of cysteine residues in its N-terminus [12, 24]. Menthol is an exception in that it activates TRPA1 by binding to the fifth and sixth transmembrane domains [17]. Ca^2+^ ions may bind to an EF hand in the ankyrin-repeat domain, although this is disputed [20, 21, 25].

Recently, the atomic structure of the human TRPA1 (hTRPA1) channel was solved, revealing channel organization, pore architecture and key regulatory interactions [26]. Like TRPV1, it has two plausible gates, an upper gate and a lower gate, along the ion conduction pathway. In contrast to TRPV1 [27, 28], however, the hTRPA1 channel structure has so far been presented only in one conformation, in which the upper gate is thought to be in a ‘partially open’ state, while the lower gate is closed [26]. The extent of upper gate opening and the role of acidic residues in the outer pore in regulating ion permeation through hTRPA1 are not yet well characterized.

In order to determine the minimum size of the TRPA1 pore in the conducting state, and to understand concentration of permeant ions by charges in an outer pore of the channel, we explored the whole-cell and single-channel properties of heterologously expressed wild-type hTRPA1 channels and with point mutations of four charged residues (D915, E920, E924 and E930) in the TRPA1 pore. The results indicate that the open channel is at least 8.2 Å in diameter at the narrowest point; they confirm that D915 is situated at the narrowest point, and they show that E920 and (to a lesser extent) E924 serve to collect permeant ions in the pore.

## Results

### Insights from the cryo-EM structure of TRPA1

**Fig 1:**
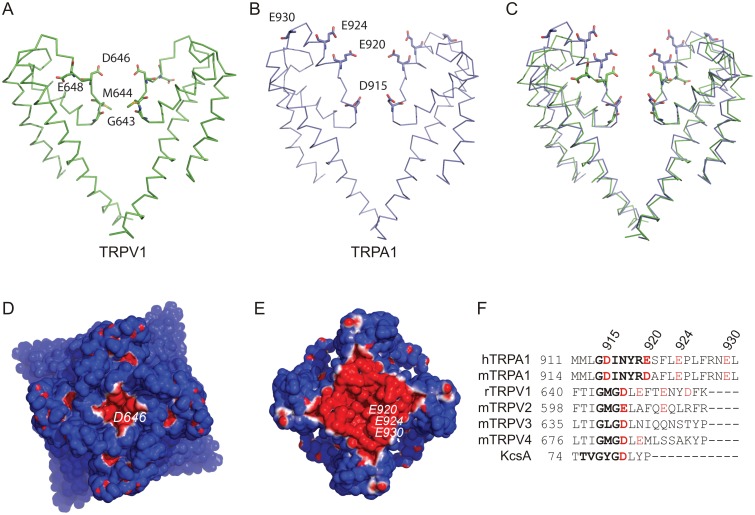
Comparison of the pores of TRPV1 and TRPA1. **A**) Cartoon representation of the pore of rTRPV1, showing S5, S6, the pore helix and selectivity filter (PDB 3J5P). The fifth and sixth transmembrane domains and the pore loop are shown for two of four subunits. Labeled residues are stick representation and colored according to atom type. **B**) Pore of hTRPA1 (PDB 3J9P). **C**) Alignment of the peptide backbones of TRPV1 and TRPA1. **D**) Surface charge representation of the extracellular face of TRPV1. The color scale in electrostatic representations is from blue (1 kT/e) to red (-1 kT/e). **E**) Extracellular face of TRPA1. **F**) Sequence alignment of TRPA1 with the TRPV subfamily and the potassium channel KcsA. Residues in bold form the pore based on published structures; those in red are acidic. The four acidic residues of hTRPA1 mutated in this study are at positions 915, 920, 924 and 930.

The atomic structure of hTRPA1 determined from cryo-EM images [26] reveals a pore domain similar to, but with some differences from that of TRPV1 [27] (Fig 1A and 1B). In a TRPV1 structure in complex with DkTx and RTX, the pore helix extending from the outer end of the fifth transmembrane domain towards the center of the pore terminates at a glycine at position 643 (G643); at the bend, M644 appears to create a constriction at the inner end of an apparent selectivity filter (Fig 1A) [27]. An acidic residue further towards the outside (D646) might attract permeant ions to the outer mouth of the selectivity filter, but another (E648) is further from the permeation pathway. In hTRPA1, a similar selectivity filter begins with D915 forming a constriction, but there are two additional acidic residues at E920 and E924 in or near the selectivity filter (Fig 1B). A third (E930) is on the outer face. An overlay of the structures (Fig 1C) shows a great deal of similarity, with more negative charge in a narrower vestibule in TRPA1. The structure-based sequence alignment is shown in Fig 1F.

Surface charge representations of the two vestibules were calculated with the Adaptive Poisson-Boltzmann Solver APBS [29] and PDB2PQR [30] tools added to PyMol (Fig 1D and 1E); these illustrate the large difference in negative charge between the two channels. In hTRPA1, the acidic residues E920, E924 and E930 create an area of ~4 nm^2^ (red) that might attract ions and increase inward current.

### Wild-type hTRPA1 outward rectification is produced by divalent cation block

**Fig 2:**
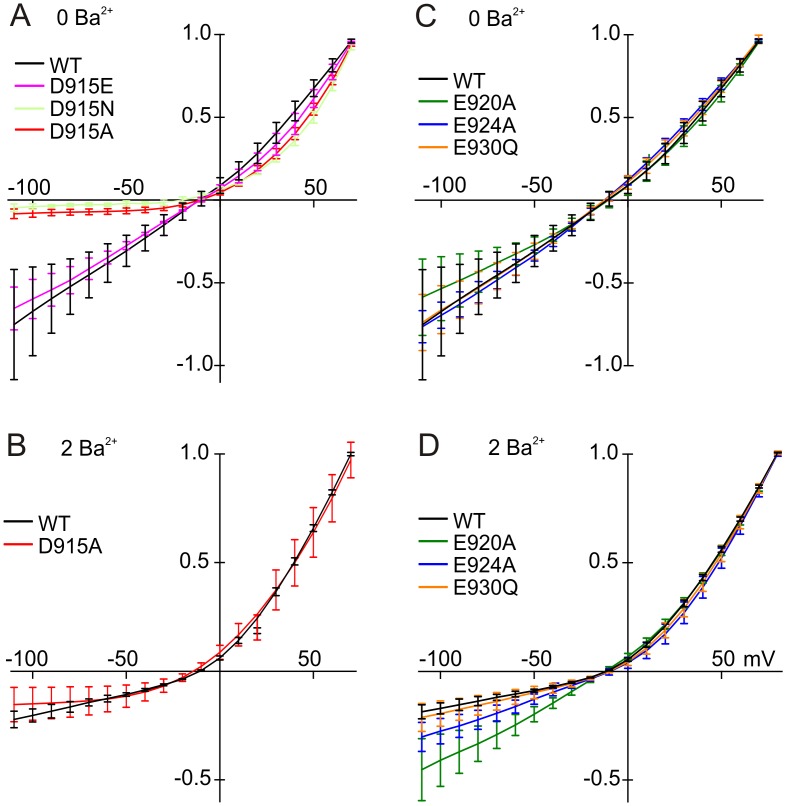
Current-voltage relationships for TRPA1 mutants with and without Ba^2+^. **A**) Mutations of D915. The wild-type I-V relation is almost linear in the absence of Ba^2+^. Charge neutralization mutations, D915N and D915A, showed greatly reduced inward current in the absence of divalent cations. The D915E mutation, which maintains the charge, was similar to wild-type. **B**) In the presence of 2 mM Ba^2+^. Inward current in wild-type was greatly reduced, but the D915A mutant was not significantly affected by Ba^2+^. **C**) Charge-neutralizing mutations of E920, E924 and E930. In the absence of Ba^2+^, the I-V relations were nearly linear for all. **D**) Wild-type TRPA1 and charge neutralization mutants with Ba^2+^. E920A was less blocked by Ba^2+^ ions than others. Curves are from an average of at least five cells measured with a voltage ramp from -115 to +85 mV. Means and standard deviations are shown. Ba^2+^ concentrations (in mM) are indicated above the panels. Currents were normalized to the current at the highest voltage.

To explore the permeation properties of hTRPA1 pore, we expressed hTRPA1 in CHO-K1 cells, activated channels irreversibly with *N*-methylmaleimide (NMM) [24] and recorded whole-cell currents. We first found experimental conditions that minimized channel desensitization in whole cell recordings and measured currents after remaining desensitization had reached steady state, usually in 2 min. For wild-type hTRPA1 in 0 mM Ba^2+^, the current-voltage (I-V) relation was nearly linear (Fig 2A). Addition of 2 mM Ba^2+^ (Fig 2B) produced substantial outward rectification in the wild-type channel, consistent with previous reports [1, 2].

Outward rectification measured in whole-cell currents could be a consequence of rectification at the single-channel level or could reflect a voltage-dependent open probability. To distinguish these, we recorded from single hTRPA1 channels, activated by NMM, in outside-out patches. The properties of these currents changed when the TRPA1 construct was mutates (see below) so we believe that the measured currents are through the expressed TRPA1 channel.

**Fig 3:**
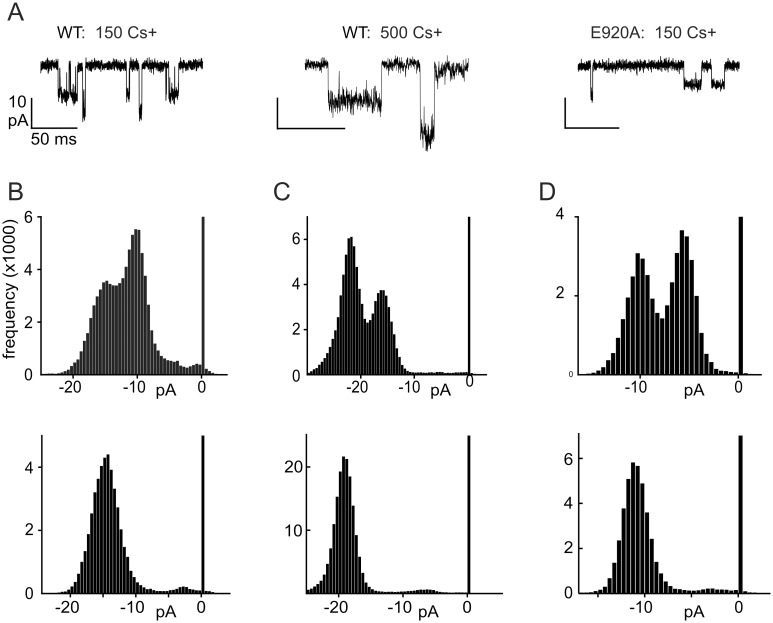
Two different open channel conductances for TRPA1. Single channel events were recorded at -60 mV. **A**) Samples of single channel recordings in a variety of experimental conditions (indicated above the representative traces). **B-D**) Frequency histograms of currents for two representative patches in each of the conditions shown in (A). One patch had openings to both conductances; a second had openings only of the larger amplitude. In each experimental condition the smaller conductance was ~60% that of the larger. Cs^+^ concentrations (in mM) are indicated above the panels. All traces in 0.1 Mg^2+^ and 0 Ba^2+^.

Single-channel records revealed two conductance states in a variety of conditions (Fig 3A), as previously reported [31]. Conductances for wild-type hTRPA1 in 150 mM symmetrical Cs^+^ and 0.1 mM Mg^2+^ at -60 mV were 251 ± 22 pS and 170 ± 15 pS (Fig 3B). These values are higher than most reported previously (66 pS, 100 pS, 140 pS [25], or 170 pS [31]), a likely consequence, for most, of the lower divalent concentration. In some recordings, the lower conductance state was rare (Fig 3B–3D, lower panels) but the larger was always present.

In 500 mM Cs^+^, the sub-state conductances were larger (345 ± 20 pS and 215 ± 34 pS; Fig 3C) but the ratio of conductances was the same. When 100 μM Ba^2+^ was added to 150 mM Cs^+^ to partially block the channel, the larger open state declined to 161 ± 11 pS and the smaller to 89 ± 10 pS (data not shown). We did not observe a flickery block, so Ba^2+^ block and unblock must be fast compared to our recording bandwidth.

We also recorded currents from the E920A mutant of hTRPA1, and saw two open states with conductances of 172 ± 13 pS and 99 ± 17 pS (Fig 3D). In every condition the smaller conductance was about 60% of the larger. Previous studies have proposed that TRPA1 undergoes a pore dilation of ~2 Å compared to a basal open state when activated by electrophiles like mustard oil in the absence of Ca^2+^ [32–34]. NMM is expected to act on the same cysteines in the ankyrin-repeat domain as mustard oil. If so, our experiments likely represent the dilated state. We did not observe a correlation with degree of channel activation. To simplify our analysis we measured only the larger open state.

**Fig 4:**
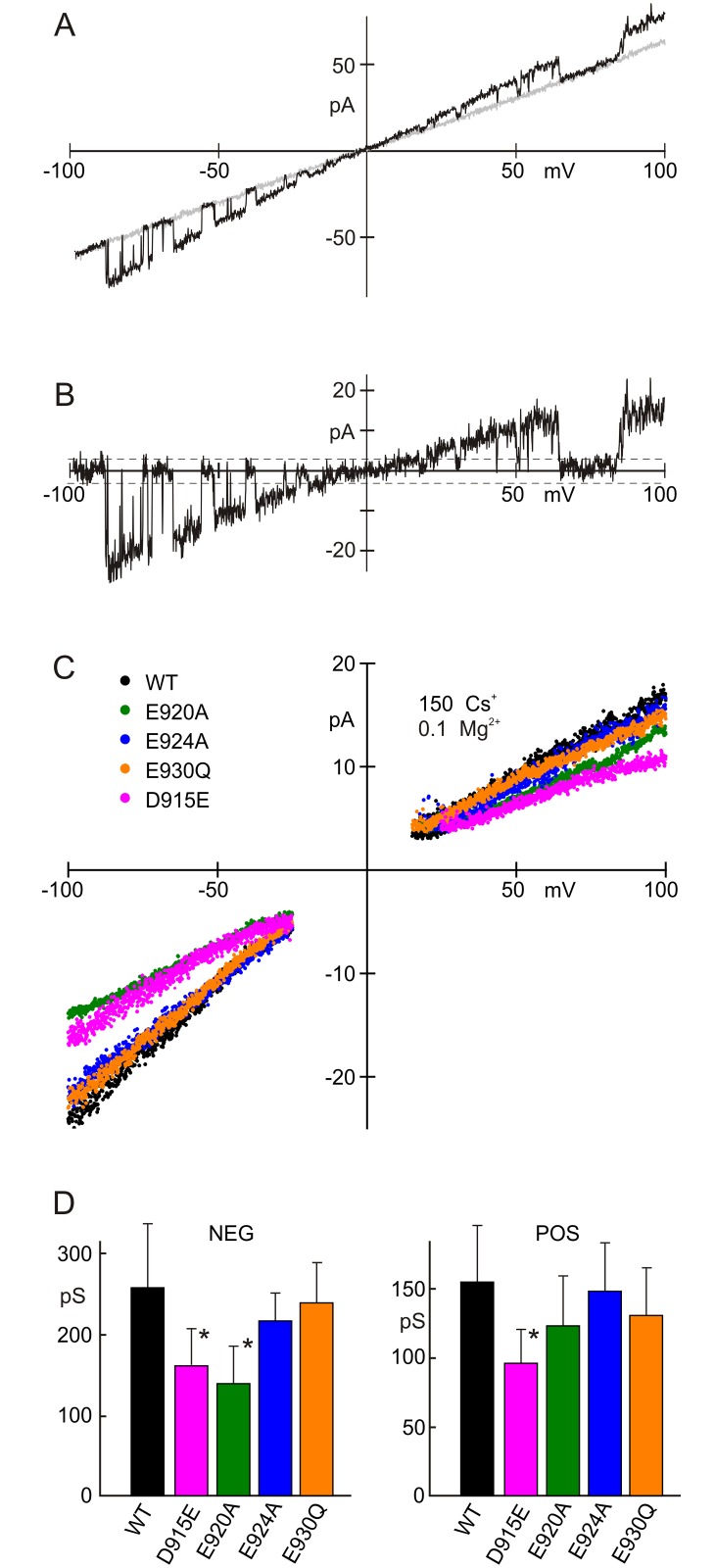
Single-channel current-voltage relationships for wild-type TRPA1 and mutants, in the absence of Ba^2+^. **A**) Sample traces of current during a voltage sweep from -100 to +100 mV; wild-type TRPA1. The black trace had one active channel and the gray (control) trace had none. **B**) Current with the control trace subtracted. The 3 pA noise threshold is indicated. **C**) Averages of open-channel current. The gap between the negative and positive sides resulted from discarding events within the noise (<3 pA). E920A and D915E have smaller conductance than wild-type at negative voltages. **D**) Single channel slope conductances for negative and positive voltages. E920A and D915E conductances are both significantly smaller than wild-type at negative potentials (Student’s T test, P<0.0005). Recorded in 150 mM symmetrical Cs^+^, 0 mM Ba^2+^, 0.1 mM Mg^2+^.

To examine rectification, single-channel I-V relations were measured with voltage sweeps from -100 to 100 mV in patches containing single TRPA1 channels (Fig 4). Control traces, with no channel openings, were subtracted from those with openings to reveal single-channel currents (Fig 4A and 4B). Open channel currents from many traces were averaged at each voltage in the sweep to produce single-channel I-V relations (Fig 4C). We found that the wild-type channel (black) is not outwardly rectified but is slightly inwardly rectified (Fig 4C). Hence, the outward rectification observed in the presence of 2 mM Ba^2+^ (Fig 2B and 2D) results from voltage-dependent block of the channel, indicating a binding site that is within the transmembrane electric field.

### Loss of negative charge at position 915 also causes rectification

In the TRPA1 structure, D915 appears as the sole residue forming the upper gate; it creates a constriction site for ion flow that is < 5Å in diameter [26]. In order to investigate the contribution of this residue to the permeation properties of TRPA1, we neutralized the negative charge at this position, with the mutations D915A and D915N. Alanine is smaller than aspartate and neutral; asparagine is the same size and polar. Both substitutions substantially reduced inward current, producing outward rectification even in the absence of divalent cations (Fig 2A). The presence of 2 Ba^2+^ did not further increase the rectification of D915A (Fig 2B).

We asked whether the extreme rectification in D915A depended on the change in residue size or residue charge. We found that the size-changing but charge-conserving mutant D915E had a current-voltage relation much like wild-type (Fig 2A). Thus it is likely that the absence of negative charge at 915, rather than a change in residue size, causes rectification, by a process independent of divalent block.

**Fig 5:**
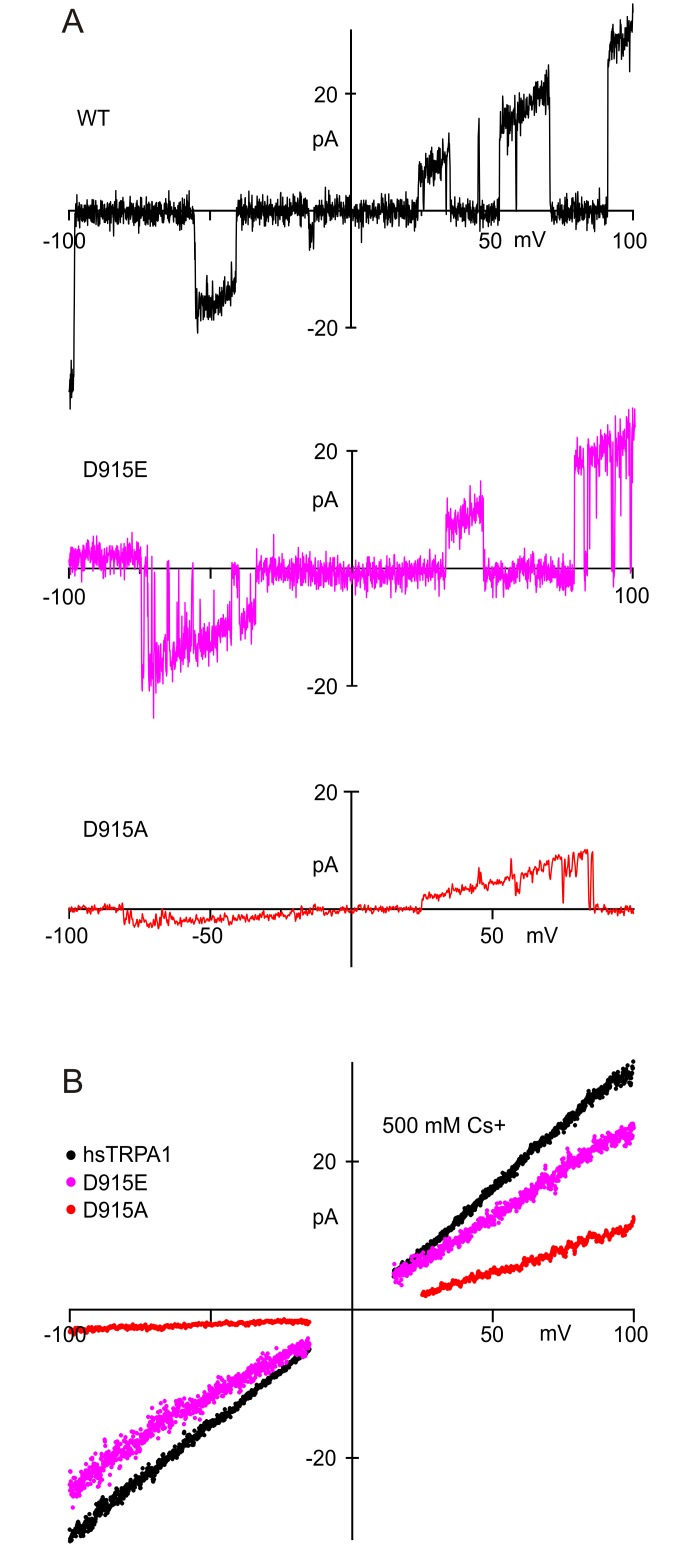
Single-channel I-V relationships forD915 mutants, in 500 mM Cs^+^. **A**) Sample leak-subtracted single-channel currents for wild-type TRPA1, and for D915A and D915E mutants. **B**) Averaged I-V relationships for wild-type TRPA1 and mutants. D915E had a slightly lower conductance at both negative and positive potentials, even in 500 mM Cs^+^. The D915A mutant was strikingly different from wild-type, with a 60% lower conductance at positive potentials and 90% lower at negative potentials. Recorded in 500 mM symmetrical Cs^+^, 0.1 mM Mg^2+^.

An alternative interpretation is that D915A affects channel gating. To investigate this possibility, we examined the single channel properties. We found that neutralization of charge by the D915A mutation significantly reduced both inward and outward single channel currents. In fact, inward single-channel currents were too small to resolve in 150 mM Cs^+^ at negative voltages, so we measured them in 500 mM Cs^+^ (Fig 5). In 500 mM Cs^+^, D915A conductance was less than wild-type at positive voltages (50% of wild-type) but much less at negative voltages (10%). The striking rectification observed in the D915A single channel currents confirms that the rectification is not due to voltage-dependent channel gating but is a pore property.

Consistent with this expectation, when we measured conductance for D915E with single-channel recording (Fig 4C), we found that the D915E mutation did not increase rectification but reduced single-channel conductance by about a third, showing a conductance of 63% and 62% of wild-type at negative and positive voltages (Fig 4D).

Although the substitution of glutamate for aspartate conserves charge, we wondered whether D915E might decrease conductance by attracting permeant ions less efficiently than D915. We measured I-V relations when Cs+ concentration was raised from 150 to 500 mM on both sides of the membrane. Ion accumulation by charge in the pore is expected to be less important at higher concentrations, but in 500 mM Cs^+^ the single-channel conductance of D915E was still significantly less than wild-type (77% and 71%) at negative and positive voltages; Fig 5B). Hence, the role of D915 is not simply attracting ions to the pore. Instead, D915 forms part of the selectivity filter and affects the energy landscape within the pore itself.

We conclude that both the charge and the size of D915 are important for permeation. The cryo-EM structure of TRPA1 suggests a pore diameter at D915 of ~7 Å, about the same as the hydrated diameter of Cs^+^, suggesting that D915 may sterically impede the passage of Cs+ and that partial dehydration is necessary for passage. The negative charge of D915 can compensate energetically for the dehydration. Eliminating charge with size unchanged (D915N) greatly reduced inward current, indicating that the charge is important to ease passage. Preserving charge but increasing size (D915E) slightly restricted current in both directions, suggesting greater steric hindrance and greater dehydration required for passage. Thus D915 apparently has both barrier and well characteristics, with the well of negative charge compensating for the steric barrier that requires dehydration.

### The pore of TRPA1 is large and D915 is the constriction site

**Fig 6:**
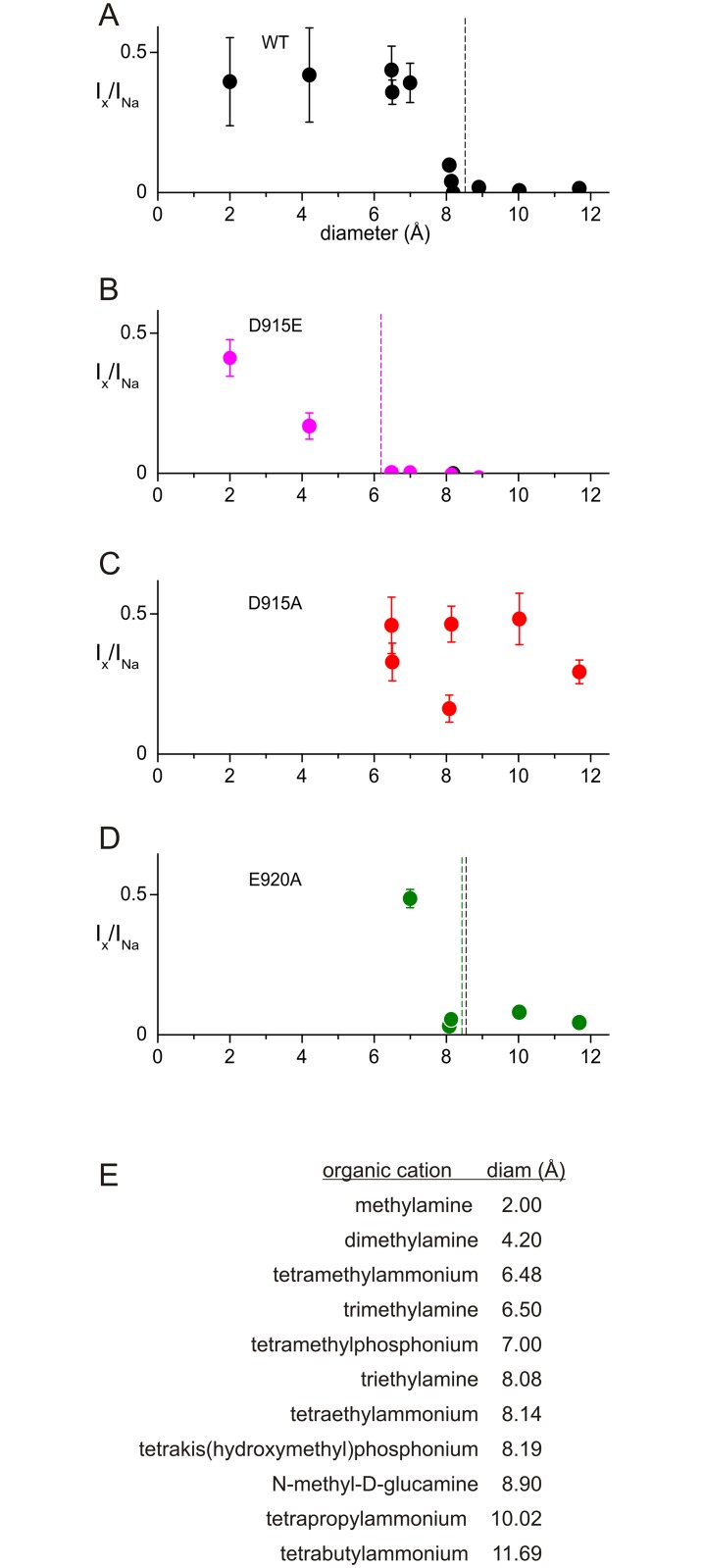
Pore size determination of wild-type TRPA1 and mutants. **A**) Organic cations of increasing size (listed in panel E) were used to determine the pore size of the wild-type TRPA1 channel. Relative permeability is plotted as the ratio of current carried by test cations to that carried by Na^+^ at -80 mV. Because some organic cations act as permeant blockers, the relative permeabilities are less meaningful than the simple cutoff where the channel no longer passed current. The minimum pore diameter of wild-type TRPA1 is estimated to be ~8.2 Å. **B**) The D915E mutant had a significantly smaller cutoff size of ~6.2 Å. **C**) D915A passed cations of at least 12 Å. **D**) The mutation of E920 to alanine did not change the cutoff size. **E**) Organic cation diameter, as calculated in Methods.

Effects of mutations suggest that D915 is a significant contributor to the energy landscape for ion permeation, most likely creating a constriction in the pore. To understand further its effect on permeation and to determine the minimum pore size of TRPA1 we used organic cations of various sizes. In some channels organic cations permeate but do not bind to the channel [35–37]. We found that some large organic cations apparently bind to hTRPA1, as evidenced by their ability to block outward current when added to the outside. Rather than calculate relative permeabilities of the cations [35], we measured the relative current at -80 mV to determine which organic cations could permeate or not (Fig 6). For the wild-type channel, organic cations including methylamine (MEA), dimethylamine (DMA), tetramethylammonium (TMA4), trimethylamine (TMA3), tetramethylphosphonium (TMP), triethylamine (TEA3), tetraethylammonium (TEA4) and tetrakis(hydroxymethyl)phosphonium (THP) were permeant, whereas larger cations such as N-methyl-D-glucamine (NMDG), tetrapropylammonium (TPA) and tetrabutylammonium (TBA) were not, suggesting a lower limit for pore diameter of ~8.2 Å (Fig 6A).

The measured pore size is thus large compared to other 6-TM-domain family members, such as KcsA (~3.5 Å) and Ca_v_ (~6 Å) [35, 36, 38–40]. The voltage-gated potassium channel blocker TEA4, with a diameter of ~8.1 Å [41, 42], showed small but measurable current through TRPA1. It is noteworthy that TRPA1 has a significantly smaller pore diameter than the hair-cell transduction channel (12.5 Å; [43]), further evidence that TRPA1 is not the molecular correlate of that channel [9].

To test the idea that D915 is the critical constriction site, we generated the D915E mutant, with the same charge but a bulkier side group. D915E has a measured minimum pore diameter of less than ~6.2 Å (Fig 6B). A decrease in pore diameter of at least 2 Å corresponds well with that expected for changing aspartate residues to glutamate residues, which if fully extended would reduce the pore diameter by 2.6 Å. The constriction by glutamate in D915E is also consistent with the decreased single-channel conductance for monovalent cations.

If D915E decreases the pore diameter, we would expect that the smaller D915A would increase diameter. Indeed it does (Fig 2C). Inward currents of D915A were small, even in divalent-free solutions. Nevertheless, we were able to distinguish the small inward current from leakage current by identifying one organic cation (TEA3, 8.08 Å) that tended to block the D915A channel and thereby could be used to set a maximum for the leak. Larger cations (TPA, 10 Å; TBA, 11.7 Å) carried more current (p<0.02).

Finally, the E920A mutant has the same cutoff (~8.2 Å) as the wild-type (Fig 6D). Thus E920 may act to collect permeant ions but is not part of the selectivity filter.

### E920 attracts permeant ions into the mouth of the pore

Negative charges in an inner or outer vestibule of an ion channel pore can affect conductance or block by attracting permeant cations into the vestibule [44, 45]. In order to investigate how the charged resides in the outer pore of hTRPA1 affect inward current, we first studied charge neutralization mutants, changing each to alanine (E920A, E924A and E930A), or creating the size-conserving but charge-neutralizing mutations E920Q, E924Q and E930Q. Here, we focus primarily on the alanine mutants, except for E930A, which did not give currents. Except as noted, we saw no significant differences between the alanine and the asparagine or glutamine mutants.

In whole cell experiments in the absence of Ba^2+^, we found that E920A, E924A and E930Q showed little rectification, like wild-type (Fig 2C). Like wild-type, E924A and E930Q were more rectified in the presence than absence of 2 mM Ba^2+^, consistent with a block of inward current (Fig 2D). E920A, however, was not blocked by 2 mM Ba^2+^, suggesting that the charge of E920 contributes to a binding site for Ba^2+^ or attracts Ba^2+^ into the vestibule. E924 and E930 are apparently more distant from the pore compared to E920, consistent with the recently reported structure.

Similarly, single-channel measurements with 150 mM Cs^+^ and 0.1 mM Mg^2+^ showed that neutralization of the charge with mutants E924A and E930Q had no significant effect on the conductance (Fig 4C and 4D). However, the single-channel conductance of E920A was significantly lower at negative voltages (54% of wild-type).

**Fig 7:**
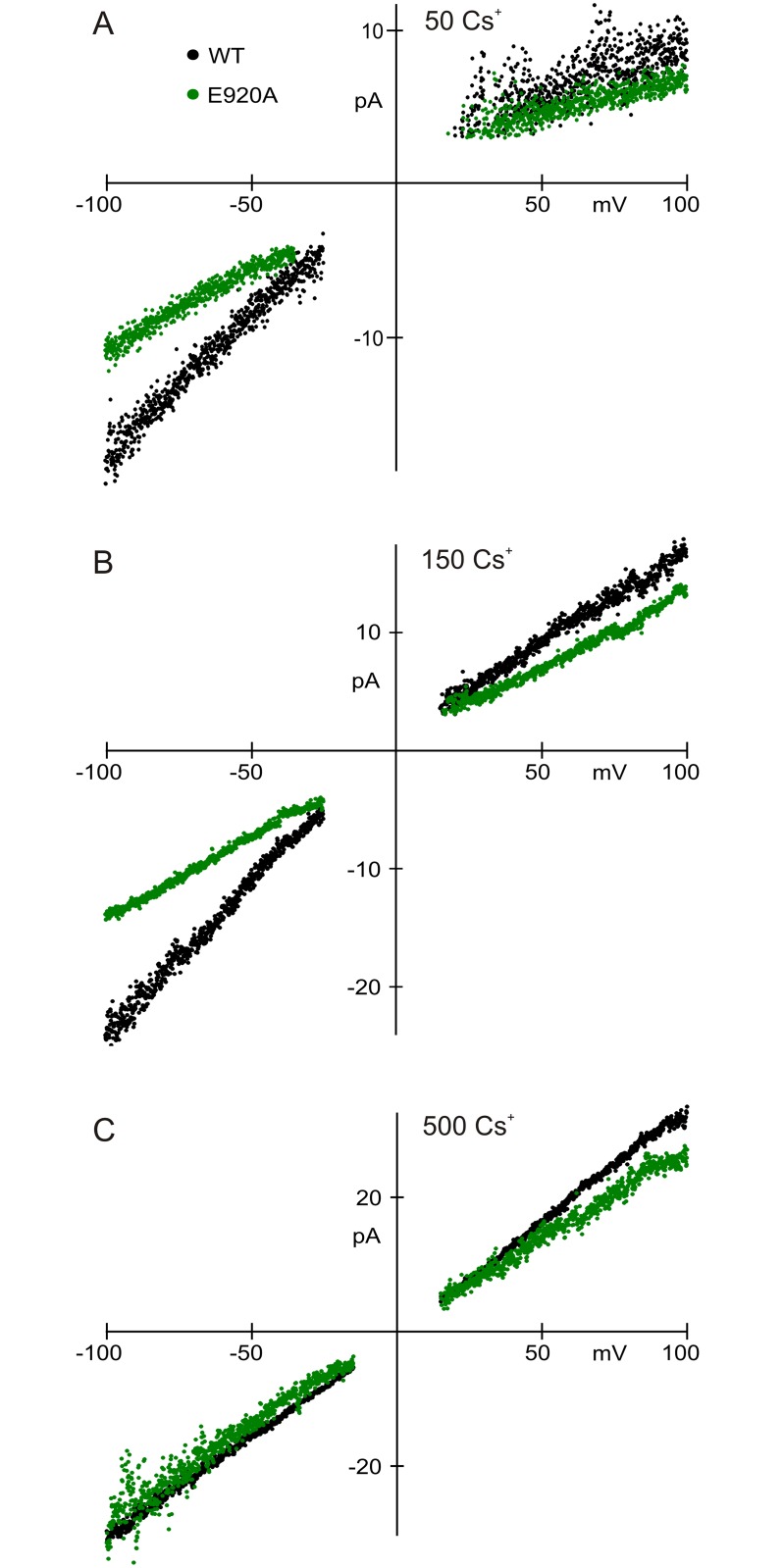
Single-channel I-V relationships for wild-type and E920A in varying concentrations of symmetrical Cs^+^ (shown in mM). An inward rectification in wild-type TRPA1 was eliminated either by the E920A mutation (**A, B**) or by high cation concentration (**C**). The slope conductance of E920A at positive voltages was less than for the wild-type channel at all concentrations, indicating that mutation of E920 to alanine also impedes outward current. Ba^2+^ concentrations (in mM) are indicated above the panels.

E920 apparently attracts permeant ions into the mouth of the pore, increasing conductance for inward currents. We tested this hypothesis by changing Cs^+^ concentration. If E920 provides important surface charge in the vestibule, then higher concentrations of Cs^+^ would screen charge, and the further neutralization of charge by E920A would have less effect. At lower Cs^+^ concentration, on the other hand, we would expect a greater difference between wild type and E920A. As predicted, in 500 mM Cs^+^ the conductances of E920A and wild-type were not significantly different at negative voltages (Fig 7C). Charge neutralization by the E920A mutation had a larger effect at 50 mM Cs^+^ (Fig 7A). At all concentrations, outward current for E920A was slightly less than for wild type, suggesting that the charge of E920 also helps draw intracellular Cs^+^ outward at positive potentials.

### Ba^2+^ interaction with hTRPA1 is also affected by charge mutants

Divalent cations both permeate and partially block TRPA1 [3, 25]. This occurs in other nonselective cation channels and has been studied extensively in voltage-gated calcium channels, which have a high affinity for Ca^2+^ and Ba^2+^ compared to sodium [40]. We tested the affinity of Ba^2+^ for hTRPA1, using concentrations of Ba^2+^ up to 30 mM. We used a constant 45-mM sodium concentration and varied the concentration of the nonpermeant cation N-methyl-D-glucamine to keep ionic strength at 145 mM.

If Ba^2+^ is a permeant blocker, the total current may decrease as Ba^2+^ concentration increases, blocking the pore, but increase again at high concentrations as Ba^2+^ becomes a significant charge carrier. This was verified in our experiments by blocking the remaining current in 30 mM Ba^2+^ with 5 μM Ruthenium Red (RuRed) (data not shown). For permeant blockers the voltage-dependent dissociation constant can be calculated by:

$$K_{D,V}=\frac{k_{-1}}{k_{1}}e^{\delta zFV/RT}+\frac{k_{2}}{k_{1}}e^{\left(\delta -\frac{1}{2}\right)zFV/RT}$$ (1)

where K_D,V_ is the voltage-dependent dissociation constant, k_1_ is the rate constant for Ba^2+^ entering the binding site in the pore, k_-1_ is the rate constant for leaving the binding site to the extracellular solution, k_2_ is the rate constant for leaving to the intracellular solution (all k’s defined at V = 0), z is the charge of Ba^2+^, and δ is the distance across the electric field to the Ba^2+^ binding site. We assume for simplicity that the energy barrier that Ba^2+^ must cross to reach its binding site is halfway from the outside to the binding site, and that the energy barrier for leaving the site to the inside is halfway from the site to the inside.

**Fig 8:**
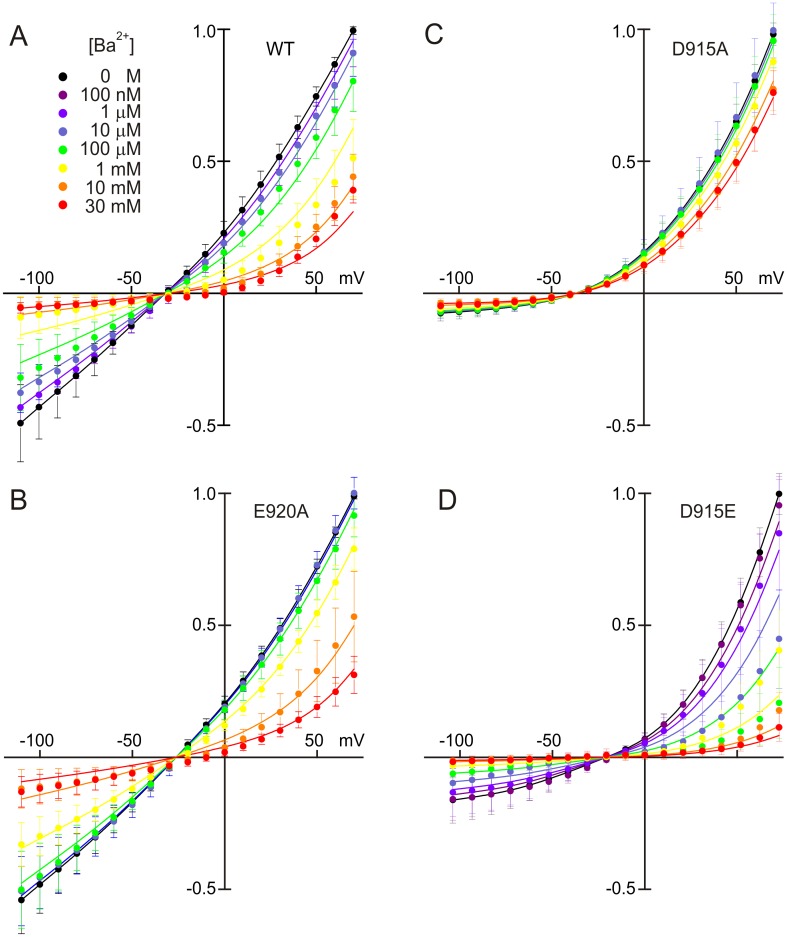
Ba^2+^ block of TRPA1 channel mutants. Normalized currents measured across a voltage range of -110 to +70 mV. **A**) Wild-type. Lines show a fit to the data using the Hill equation with a voltage-dependent dissociation constant (Eq 1). **B**) Block by Ba^2+^ of the E920A channel. E920A had less block than wild-type. **C**) D915A showed little block by Ba^2+^, however this result was complicated by the unblocked rectification of the channel produced by the mutation. **D**) Block of the D915E mutant by Ba^2+^; affinity was three-fold greater than wild-type. N = 6; mean+SD. Fitting parameters are in S1 Table.

Fig 8 shows current-voltage relations for different Ba^2+^ concentrations, fitted with the Hill equation using the dissociation constant of eq 1. The best fit for the wild-type channel (Fig 8A) has a K_D_ of 0.6 mM at 0 mV (S1 Table). The K_D_ changes little at negative potentials, indicating that the voltage-dependence of Ba^2+^ entering from the outside is approximately matched by the voltage dependence for passing through to the cytoplasm. E920A has three-fold lower affinity for Ba^2+^ (Fig 8B) and D915A has an order of magnitude less affinity than wild-type (Fig 8C and S1 Table). All the fits indicated that δ is approximately 0.5, suggesting that Ba^2+^ binds about halfway across the electric field. Hill coefficients range from 0.34 to 0.63 (S1 Table).

If the pore region is highly negatively charged, this would increase the concentration of cations—both permeant cations as well as blocking cations—over the concentration in bulk solution (reviewed in [44]). However as the bulk concentration of cations increases, they would screen the negative charge and its concentrating effect would decrease. This could explain a Hill coefficient less than 1. Neutralizing E920 with alanine produced a Hill coefficient much closer to 1, supporting the idea that this residue plays an important role in attracting ions into the mouth of the pore.

The D915E mutant had a smaller single-channel conductance than wild-type, suggesting that the pore is smaller than in wild-type. We might then expect this channel to have a higher affinity for divalent cations as charges in the pore might more closely coordinate a blocking cation. Indeed, the affinity of D915E for Ba^2+^ was three-fold higher than for wild-type (Fig 8D and S1 Table). Apparently, both D915 and E920 participate in creating a binding site for Ba^2+^, but the greater dependence of block on charge at D915 suggest the binding site is near the lower residue, and the oxygen on N917 may participate in coordinating divalents.

### Charged pore blocker affinity is diminished in both E920A and D915A

**Fig 9:**
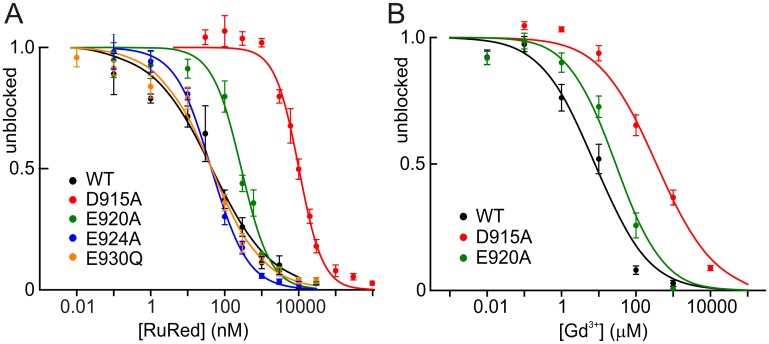
Block of TRPA1 channels by charged pore blockers. Block is indicated as a fraction of the unblocked current. **A**) Block of TRPA1 and mutants by Ruthenium Red. **B**) Block by gadolinium. D915A had a much lower affinity for both blockers, and E920A had a smaller but significant decrease in affinity. N> 6; mean and standard error of mean (SEM) are shown.

The effects of these mutations on conductance suggest that D915 forms the narrowest part of the pore, and that E920 is near or within the pore and helps collect both permeant ions and blockers—both consistent with the cryo-EM structure [26]. To further test this, we asked if pore-blocking molecules blocked charge-neutralizing mutants less well. RuRed, which blocks many TRP channels, is a hexavalent cation and therefore should be very sensitive to the charge in the mouth of the pore. RuRed blocked the E930Q and E924A mutants just as well as the wild-type hTRPA1 (Fig 9). However it blocked E920A six-fold less well and D915A 200-fold less well (S2 Table). We also observed that the Hill coefficients for E920A and D915A were ~1, but the wild-type channel had a Hill coefficient of 0.54. This may be explained if surface charge provided by E920 and D915 concentrates this highly charged blocker near the pore, an effect discussed further below.

We also used gadolinium block to test the charge neutralization mutants. Gadolinium is a trivalent cation and—unlike RuRed (9.7 x 9.7 x 13.2 Å)—its charge is not spatially dispersed. The Hill plots (Fig 9) show that the block of hsTRPA1 by gadolinium is also affected by charge neutralization, but not as greatly as for RuRed. This is expected given the lower charge of gadolinium. E920A’s affinity for gadolinium is 4-fold lower than wild-type and D915A’s affinity is over 10-fold lower (summarized in S2 Table). The effect of the E920 and D915 mutants on blocker affinity suggest these residues are in or very near the blocking site, and that E924 and E930 are not, again consistent with the structure.

## Discussion

Ion permeation and channel gating start at the extracellular surface of the ion channel proteins, where charged residues may act to attract permeant ions (and blockers) into their conduction pores [45–47]. We find that in TRPA1, residue E920 and to a lesser extent E924 serve this function. The Hill coefficient of <1 for RuRed and gadolinium block is consistent with a highly charged pore mouth of TRPA1. Neutralizing E920 reduced block and brought the Hill coefficient closer to 1 for these blockers and for Ba^2+^.

We can use blocker affinity to calculate the change in electrostatic potential at the blocking site caused by the E920A mutation, because the ratio of the blocker dissociation constants varies with both the charge of the blocker, z, and the change in the surface potential Δφ at the blocking site [46]:

$$\frac{K_{D,mut}}{K_{D,WT}}=e^{-zF\Delta \emptyset /RT}$$ (2)

Calculated values for the change in surface potential caused by the E920A mutation are 15.5 mV for Ba^2+^ and 10.9 mV for Gd^3+^.

The dependence of conductance on ion concentration is further evidence that E920 is an important surface-charge residue. Higher ion concentrations screen the exposed charges of a protein, reducing their ability to concentrate ions. Therefore the conductance of channels with and without charges should converge at higher permeant ion concentrations, as we find for wild-type and E920A single-channel conductance (Fig 7). We can also estimate the change in surface potential from the decrease in single channel conductance in E920A. Ion concentration at a site in the pore should vary with the electrostatic potential by:

$$\left[I\right]={\left[I\right]}_{b}e^{-zF\emptyset /RT}$$ (3)

where [I]_b_ is the ion concentration in bulk solution and z is its charge. Assuming that conductance is proportional to permeant ion concentration in the mouth of the pore, the ratio of conductances similarly depends on surface potential. This predicts a Δφ for E920A of 16.0 mV, which agrees well with that found for Ba^2+^ block. That the potential change for Ba^2+^ block is the same as that for conductance suggests that Ba^2+^ blocks at the limiting site for permeation.

Our measurements show that the glutamate at position 920 plays an important role in collecting cations into the pore, making the effective surface potential by ~16 mV more negative, but acidic residues further out have little effect on permeation. Is this consistent with prediction from the spatial arrangement of charge in the cryo-EM structure? The Debye-Hueckel theory gives the distribution of potential ϕ around a central ion of valence z free in solution as:

$$\varphi =\frac{zqe^{-\kappa r}}{4\pi \varepsilon_{0}\varepsilon r}$$ (4)

where q is the elementary charge, r is the radius, 1/κ is the characteristic decay constant (inverse of Debye screening length), ε is the relative permittivity, and ε_0_ is vacuum dielectric permittivity. With previously estimated constants for a charge in electrolyte solution (as in ref [48], page 546), the quantity *q*/4*πε*_0_*ε* can be approximated as 180 mV/Å. When the charge is at the interface of an electrolyte solution and a low-dielectric constant medium such as lipid or protein, the potential approximately doubles, since the charge cannot be neutralized by water on one side [48, 49]. With these assumptions, a charge that is about one Debye length away (~8 Å for electrolyte solution of ~150 mM salt concentration) produces a local potential of 8–16 mV. In the cryo-EM structure, a centrally located cation near D915 would be about ~11 Å away from each of the E920 side chain oxygen atoms. With four negative charges around the vestibule in a tetramer and assuming linear summation, this would add to approximately 16 mV at the center of the pore near D915, consistent with what our measurements suggest.

**Fig 10:**
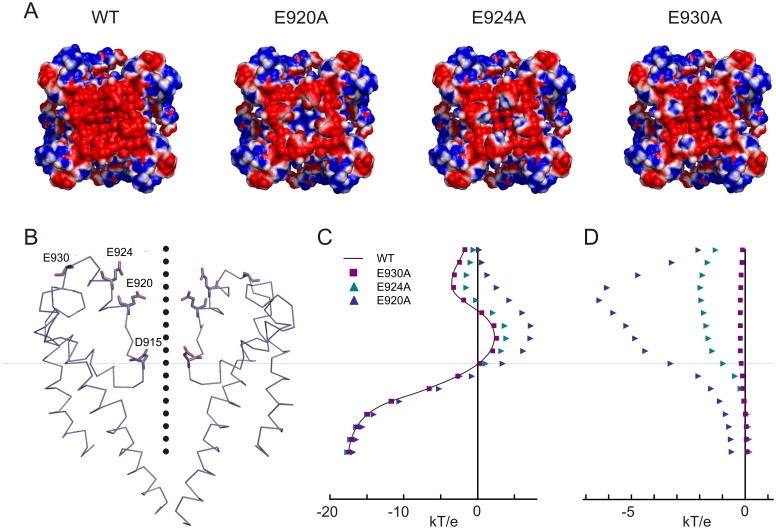
Electrostatic contributions of acidic residues in the vestibule. **A**) Surface charge representation of hsTRPA1, viewed from outside the cell, and calculated effect of alanine substitutions. Shown are solvent-accessible protein surface areas, color-coded according to electrostatics (blue to red represents +25.4 to -25.4 mV or +1 to -1 kT/e). E920 is critical for producing a negative potential in the vestibule. E924 and E930 have less influence in the vestibule. **B**) Cartoon structure of the TRPA1 pore domain, with alanine-substituted residues shown in stick mode. Black dots, spaced at 1.2 Å, indicate positions at which electrostatic potential in the permeation pathway was calculated. **C**) Electrostatic energy profile for the positions in B and effect of alanine substitutions. **D**) Energy contributions of E920, E924 and E930, calculated as difference from wild-type.

We also used the Adaptive Poisson-Boltzmann Solver tool [29] to calculate the electrostatic surface potential for the wild-type and each of the mutant channels (E920A, E924A and E930A), assuming 150 mM monovalent salt. Fig 10A shows the surface representations viewed from the extracellular side. The local increase in electrostatic potential due to each mutation is evident in these plots, with blue indicating more positive potential. We then calculated the electrostatic potential along the permeation pathway for each mutant in the absence of a transmembrane potential (Fig 10B and 10C), and calculated the contribution of each residue to the potential by subtracting the mutant profile from the wild-type (Fig 10D). Near residue D915, the contribution of E920 is in the range of -3 to -4 kT/e or -75 to -100 mV. The change in potential near D915 contributed by E924 is about -25 mV (Fig 10D). These significant changes in electrostatic potential with mutation are qualitatively consistent with potentials calculated from Ba^2+^ block, however the values are about five-fold higher. The difference may be due to limitations and assumptions in the computational modeling approach (see Methods). It is also possible that if the outer gate opens wider, as we expect from the minimum diameter determined from large permeant organics, the separation of charged residues will reduce the electrostatic potential in the center of the pore. Nevertheless, the calculations show the relative contribution of different acidic side chains to an electrostatic potential that would attract both permeant and blocking ions.

The second step of ion permeation is passage through the selectivity filter. Here, waters of hydration are at least partially stripped from the permeant ion, compensated by negatively charged or polarized atoms from the protein. In potassium channels these are a series of backbone carbonyl oxygens from the pore sequence TVGYGD [39]. Surprisingly, the crystal structure of a nonselective bacterial channel (NaK) showed a similar organization of carbonyl oxygens chelating the permeant ion [38]. On the other hand, in sodium and calcium channels the side chains of the pore residues are thought to line the pore and bind the permeant cation [50, 51].

What happens in TRPA1? The physiology suggests interaction of the residues with permeant ions. The D918A mutation in mTRPA1 (orthologous to D915 in hTRPA1) significantly decreased the calcium permeability; D918E increased it [25]. Wang et al. argued that the D918 side chains do not directly coordinate divalent cations but stabilize at a distance by electrostatics. If only electrostatic interaction mattered, however, aspartate and glutamate—having the same charge—should interact almost equally with permeant ions. Another possibility is that the mutation of D918 to glutamate displaces the carbonyl oxygens of the peptide backbone, indirectly altering the structure of the pore domain to increase the calcium affinity, as was suggested for the A66E mutation in the NaK channel [52]. Our experiments suggest instead that the D915 side chain of TRPA1 interacts directly with the permeant ion rather than being buried in the protein. The observed >2 Å decrease in pore diameter in D915E, measured with permeant organics, matches the 2.6-Å change expected if aspartate or glutamate side chains at position 915 extend fully into the pore. Mutating aspartate to the larger glutamate might distort the pore if the side chain is buried within the protein, but probably not as much as 2 Å, since only a small change in the selectivity filter structure was seen in the case of the NaK channel mutant D66E [52]. Indeed, the cryo-EM structure is consistent with the side chain of D915 facing the pore and constituting the tightest restriction site.

The cryo-EM structure shows an outer gate that measures ~7 Å between diagonally opposed D915 residues. However, given that the side chains cannot be fully resolved at the resolution of the available structure, and given that the structure may not be representative of an open conformation, there is an uncertainty to this estimate. Our estimate of at least 8.2 Å is based on current carried by a panel of organic cations. Kawashimi, testing similar organic cations, instead estimated a pore diameter of 13.5 Å [32]. Their figure was based on extrapolation from the largest cation they tested (NMDG, 8.9 Å), using reversal potentials and the excluded volume method. An estimate based on inward current can differ from that using reversal potential if the permeant ion binds within the pore; the very small current we observed with NMDG suggests this is the case. Bobkov et al. also observed a low but detectable permeability for NMDG [31]. The difference between their estimated pore diameter (7.4 Å) and ours can be attributed to differing methods of measuring molecular dimensions. Thus a wild-type pore diameter of 8–8.5 Å is most likely.

At the same time, some larger organic cations can pass through TRPA1. FM1-43, a fluorescent styryl dye (10.5 Å), passes through both the TRPV1 and TRPA1 channels [53]. Gentamicin conjugated to Texas Red can also pass through TRPA1 [54]. Texas Red, a fluorescent dye with a rigid planar ring structure, is at least 11.5 Å across. However the assays are not really comparable: a measurable current requires millions of ions per second, whereas detectable fluorescence can result from thousands of molecules accumulated over minutes. It is perhaps not surprising that organic molecules larger than the nominal pore size could occasionally slip through. X-ray crystal structures of potassium channels show that the peptide backbone of the selectivity filter is not rigid but moves to coordinate potassium ions [55] and molecular dynamics simulations of ion permeation through TRPV1 reveal considerable movement of both backbone and side chains on a time scale of nanoseconds [56]. It is not impossible to imagine the pore briefly distending to allow a large organic to pass.

Overall, these data suggest the following view, largely consistent with the cryo-EM structure: E920 is located in the outer mouth of the pore and acts to collect ions to the mouth, but E924 and E930 are too distant to affect permeation much. D915 lines the pore and represents the constriction point of the channel, which is consistent with the recently reported structure of TRPA1. We find that the constriction measures at least 8.2 Å, large compared to most ion channels and slightly larger than the ~ 5 Å estimate that is based on the reported structure. As suggested by Paulsen et al., the upper gate in the structure (D915) likely represents not a fully open but rather, a partially closed channel conformation.

This model could inform a search for selective antagonists of TRPA1, pointing towards highly charged molecules that could be attracted to the mouth of pore by E920 and also bind at D915. If the largest diameter of a charged blocker was larger than the minimum pore diameter of ~8.2 Å but included a smaller chain with a charge on the end, this molecule might selectively bind in the pore of TRPA1 but not in similar cation channels, eliminating off-target effects.

## Materials and Methods

### Molecular Biology

The hTRPA1 construct in the pFROG3 mammalian/oocyte expression vector was kindly provided by Dr. David Julius. The GFP construct was created from the IRES eGFP cassette from pIRES2-EGFP cloned into the pcDNA3.1 vector. Point mutations were introduced using the QuikChange Site-Directed-Mutagenesis kit (Stratagene). Point mutations were designed to create or destroy an enzymatic restriction site, to verify that the correct point mutation was created by analytical digest, and were further verified by sequencing.

### Cell Culture

CHO-K1 cells from ATCC were maintained in Ham’s F12K modified serum with 10% FBS and 1% penicillin/streptomycin (Invitrogen). Human TRPA1 wild-type and mutant constructs were transiently transfected into CHO-K1 cells at confluency using lipofectamine 2000 (Invitrogen). GFP was cotransfected with the hsTRPA1 constructs at a 1:3 ratio to mark transfected cells. Six to eight hours after transfection, the cells were trypsinized, triturated, and replated onto a protamine-treated (1 mg/ml) 18-mm coverslip. Medium contained 5 μM ruthenium red to block TRPA1 channels that were open at rest and would otherwise lead to cell death. Cells were used for electrophysiology 24–48 hours post-transfection.

### Electrophysiology

Pipettes for whole-cell recording were R6 glass (1.65 mm OD, 1.20 mm ID with a filament; King Precision Glass) pulled to 2–5 MΩ resistance with a Flaming/Brown puller (P-87, Sutter Instruments). Pipettes for single-channel outside-out patch recording were KG-33 glass, pulled to 6–10 MΩ. Both were polished with a microforge, and painted with dental wax (Hygenic Corporation) to decrease capacitance. The Axopatch 200B amplifier had its low-pass Bessel filter set to 5 kHz (except 1 kHz for D915A single-channel recordings). The stimulus waveform was controlled by pClamp 9.0 software (Axon Instruments) with a sampling rate of 10 kHz and responses digitized using a Digidata 1322A (Axon Instruments). A voltage-ramp protocol changed the voltage from -100 mV to 100 mV in 200 ms. For whole-cell data, each cell was corrected for whole-cell capacitance and series resistance was compensated to 80% with a lag of 10 μs.

### Data analysis

Data were analyzed in Microsoft Excel. The liquid junction potential was corrected offline. For whole-cell data analysis, an average of five sweeps was acquired before a solution change to establish the control current and five sweeps were averaged during equilibrium after solution exchange. For single-channel data, the control trace representing leak conductance was the sweep with no channel opening occurring closest in time to experimental sweeps that contained only one channel opening. Noise was filtered out by removing any current points below 3 pA in magnitude, and records from multiple sweeps and patches were averaged to get single-channel current-voltage (IV) relations. For the single channel histograms, the data were analyzed using the Clampfit 9.2 single-channel-search event detection.

### Solutions

For most whole-cell experiments the control extracellular solution was (in mM): 140 NaCl, 5 CsCl, 2 BaCl_2_, 1 MgCl_2_, and 10 HEPES; pH adjusted to 7.4 with NaOH, and osmolarity adjusted to 330–340 mOsm with sucrose. The normal pipette solution was (in mM): 140 aspartic acid, 10 NaCl, 4 MgCl_2_, 5 BAPTA, 10 HEPES, 4 Na_3_ATP, 0.3 Li_3_GTP, and 0.1 CaCl_2_; pH adjusted to 7.4 with CsOH, and osmolarity adjusted to 315–325 mOsm with sucrose. Ba^2+^ in external solutions minimized desensitization, and we took measurements after remaining desensitization had reached steady-state, typically after 2 min. The shapes of all current-voltage relations were the same at the peak conductance and the desensitized conductance. To test various divalent cations on desensitization, 2 mM Ba^2+^ was replaced by Mg^2+^, Ca^2+^, or Sr^2+^. For current-voltage relations with no divalent ions present, the solutions were (in mM): extracellular—140 NaCl and 10 HEPES at pH 7.4 and 330–340 mOsm; intracellular—130 aspartic acid, 10 CsCl, 5 BAPTA, and 10 HEPES at pH 7.4 and 315–325 mOsm.

For single channel experiments, external and internal solutions were symmetrical except in the internal solution 10 mM F^-^ replaced 10 mM Cl^-^. The solutions were 490 mM CsCl, 140 mM CsCl or 40 mM CsCl, with 10 mM HEPES and 0.1 mM MgCl_2_ (except in 40 mM CsCl which had 0.01 mM MgCl_2_). pH was 7.4 and osmolarity was unadjusted. Pore blockers were added from stock solutions of 5 mM for ruthenium red or 1 M for GdCl_3_. For barium-block experiments solutions all contained 45 mM NaCl, BaCl_2_ as indicated, 10 mM HEPES, and NMDG as needed to maintain 145 mM ionic strength. pH was adjusted to 7.4 with HCl and osmolarity was 330–340 mOsm. For organic cation experiments, solutions were of the general form (in mM): 140 XCl, 10 HEPES, pH adjusted with XOH, and 330–340 mOsm, where X is methylamine, dimethylamine, tetramethylammonium, trimethylamine, tetramethyl-phosphonium, triethylamine, tetraethylammonium, tetrakis(hydroxymethyl)phosphonium, N-methyl-D-glucamine, tetrapropylammonium, or tetrabutylammonium. Intracellular solution was the same as for divalent-free conditions. Channels were activated using 50 μM *N*-methylmaleimide, dissolved from a 10 mM stock and applied for 20 s. Cells were superfused using a ValveLink 8 perfusion system (Automate Scientific).

### Determination of Organic Cation Size

Organic cation structures were constructed in ChemDraw Ultra 9.0 and the energy minimized, then imported into the Visual Molecular Dynamics (VMD) program (Theoretical and Computational Biophysics Group, University of Illinois). VMD was used to measure the distance between atoms of each vertex and the atomic radii were added to get the overall distance between vertices. The average of all distances was used for the diameter.

### Determination of Electrostatic Potential

TRPA and TRPV atomic structures (PDB 3J9P and 3J5Q, respectively) were used as inputs. Mutations were introduced to TRPA structure in silico using PyMOL [57] and electrostatics were calculated using the APBS and PDB2PQR tools, at http://www.poissonboltzmann.org/ [29]. The ‘solvent radius’ to probe the surface of the protein was set to the default value of 1.4 Å, which approximates the radius of a water molecule. Additional APBS utilities were used to determine the potential in the channel pore by solution of the linearized Poisson-Boltmann equation in the absence of lipids with a solute dielectric of 2 and solvent dielectric of 78.5 in the presence of 150 mM monovalent salt. PyMOL [57] was used for all visualizations. We note that the exact values for the solute dielectric constants are not well determined [58] and they have some bearing on the output, adding to uncertainty of the estimates (data not shown). The color scale in electrostatic representations is from blue (1 kT/e) to red (-1 kT/e).

## Supporting Information

S1 Table Fitting parameters for block of hsTRPA1 by barium. See main text for explanation of eq 1.
(PDF)

S2 Table Block of hsTRPA1 by Ruthenium Red and gadolinium. (PDF)
