# Supplementary material for: The Outer Pore and Selectivity Filter of TRPA1
Source: PLoS One. 2016 Nov 8;11(11):e0166167. doi: 10.1371/journal.pone.0166167 (PMC5100928; doi:10.1371/journal.pone.0166167)
Supplement: S1 Table — See main text for explanation of eq 1. (PDF) [file pone.0166167.s001.pdf]

Construct	$K_{D,0\ mV}$	δ	n	k_{-1}/k_1	k_2/k_1
WT	0.6 ± 0.01	0.51 ± 0.001	0.39 ± 0.001	0.063 ± 0.001	0.58 ± 0.01
E920A	2 ± 0.01	0.50 ± 0.001	0.63 ± 0.002	0.15 ± 0.003	1.9 ± 0.01
D915A	6.1 ± 0.06	0.54 ± 0.001	0.34 ± 0.002	0.021 ± 0.008	6.1 ± 0.05
D915E	0.23 ± 0.006	0.49 ± 0.003	0.36 ± 0.002	0.011 ± 0.001	0.21 ± 0.005
