# Supplementary material for: The Outer Pore and Selectivity Filter of TRPA1
Source: PLoS One. 2016 Nov 8;11(11):e0166167. doi: 10.1371/journal.pone.0166167 (PMC5100928; doi:10.1371/journal.pone.0166167)
Supplement: S2 Table — (PDF) [file pone.0166167.s002.pdf]

Construct	RuRed IC50 (nM)	RuRed n	Gd^{3+} IC50 (μM)	Gd^{3+} n
wt	45.2	0.54	7.89	0.71
E930Q	43.5	0.62		
E924A	44	0.88		
E920A	281	1.11	28.3	0.86
D915A	10100	1.35	354	0.66
